# Incidental Finding of a Large Mobile Aortic Arch Mass during Conventional Angiography

**Published:** 2017-10

**Authors:** Hassan Aghajani, Shahrooz Yazdani, Mohammad Ghafaripoor, Zahra Shajari

**Affiliations:** 1 *Tehran Heart Center, Tehran University of Medical Sciences, Tehran, Iran.*; 2 *Clinical Research Development Unit, Shahid Rajaei Hospital, Alborz University of Medical Sciences, Karaj, Iran.*

**Keywords:** *Aorta, thoracic*, *Thromboembolism*, *Angiography*

## Abstract

Thromboembolism occurs commonly in general practice and leads to significant health burden. Apart from cardiac sources, aortic atherosclerotic plaques contribute considerably to thromboembolism. A 63-year-old diabetic hypertensive woman referred to our center due to exertional chest pain unresponsive to optimal medical therapy and underwent coronary angiography. Owing to resistance during guide-wire advancement, an aortography was performed. Aortic arch injection demonstrated a large suspended mass distal to the left subclavian artery with free movement in the descending thoracic aorta. Echocardiography revealed widespread atherosclerotic changes in the aortic arch with a large hypermobile mass. Dual-source multi-slice (2 × 128:256) computed tomography angiography of the whole aorta revealed a large floating mass (in favor of a thrombus) in the distal portion of the arch. The patient underwent coronary artery bypass grafting due to severe coronary artery disease. The intra-aortic mass, which was actually a large atherosclerotic plaque, was resected at the same session. She was discharged uneventfully and during a 1-year follow-up, she had no embolic events.

## Introduction

Thromboembolism is a leading cause of morbidity and mortality. Cardiac disease is the most universal source of arterial emboli and accounts for approximately 80% of all cases.^1^ Ulcerated atherosclerotic plaques in the aorta and carotid arteries are important noncardiac sources of emboli. Embolization of thrombi in the aorta can also be associated with cancer, pregnancy, and hypercoagulable states.^2^^-^^5^ Finding of a floating mass in the aortic arch is rare and the management remains controversial. Herein, a case of a large floating atherosclerotic plaque in the aortic arch is reported.

## Case Report

**Figure 1:**
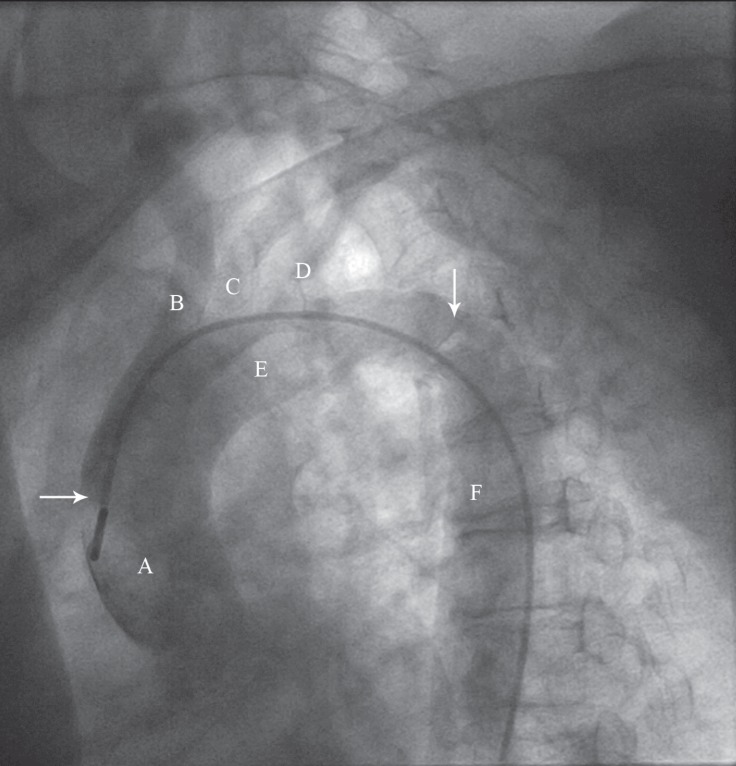
Aortogram in the left anterior oblique (LAO) projection reveals a mobile mass in the aortic arch distal to the left subclavian artery. Ascending aorta is shown with letter A. Innominate, left common carotid, and left subclavian arteries are shown with letters B, C, and D, respectively. Letters E and F show the aortic arch and the descending thoracic aorta, respectively. Aortic mass is marked with the vertical arrow. Pigtail catheter in the ascending aorta is marked with the horizontal arrow.

A 63-year-old diabetic hypertensive woman was admitted to our center due to exertional chest pain unresponsive to optimal medical therapy. Coronary angiography was planned to define the extent of coronary artery disease. The patient had an outpatient transthoracic echocardiography (TTE), which reported almost normal indices. Conventional coronary angiography was initiated with the advancement of a 0.035 soft J-tipped wire. During the advancement of the wire in the descending thoracic aorta and the aortic arch, some resistance was felt and the wire was passed to the ascending aorta with gentle manipulation. The patient had significant stenosis in the left main stem and the major epicardial coronary arteries. Before the withdrawal of the wire from the ascending aorta, a pigtail catheter was placed in the aortic arch to obtain an aortogram. Aortography revealed a large floating mass distal to the left subclavian artery with free movement in the descending thoracic aorta (Figure 1, Movie 1). 

Differential diagnosis at this time included dissection (iatrogenic or spontaneous), mural thrombus, tumors, and atherosclerotic plaque. The patient had had no febrile illness recently and also reported no constitutional symptoms including weight loss. Complete blood count revealed no leukocytosis. Inflammatory markers including erythrocyte sedimentation rate and C-reactive protein were within the normal ranges. In addition, antinuclear antibody and rheumatic factor were negative. Renal function was normal, and fasting blood sugar was 162 mg/dL. Other laboratory findings were within the normal ranges.

**Figure 2:**
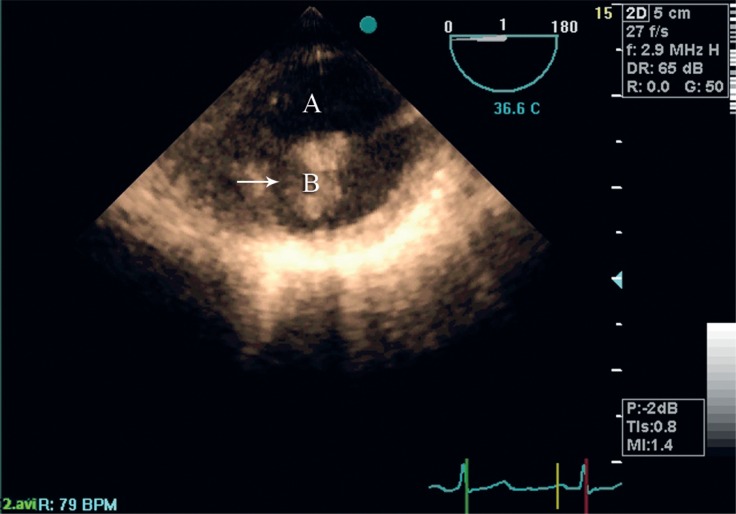
Transesophageal echocardiography (TEE) reveals a large mobile mass in the aortic arch. TEE angle at 0° shows a cross-section of the aorta. Letter A stands for the internal lumen of the aortic arch. Letter B shows a large atherosclerotic plaque. Horizontal arrow shows the attachment site of the atherosclerotic plaque to the aorta.

**Figure 3:**
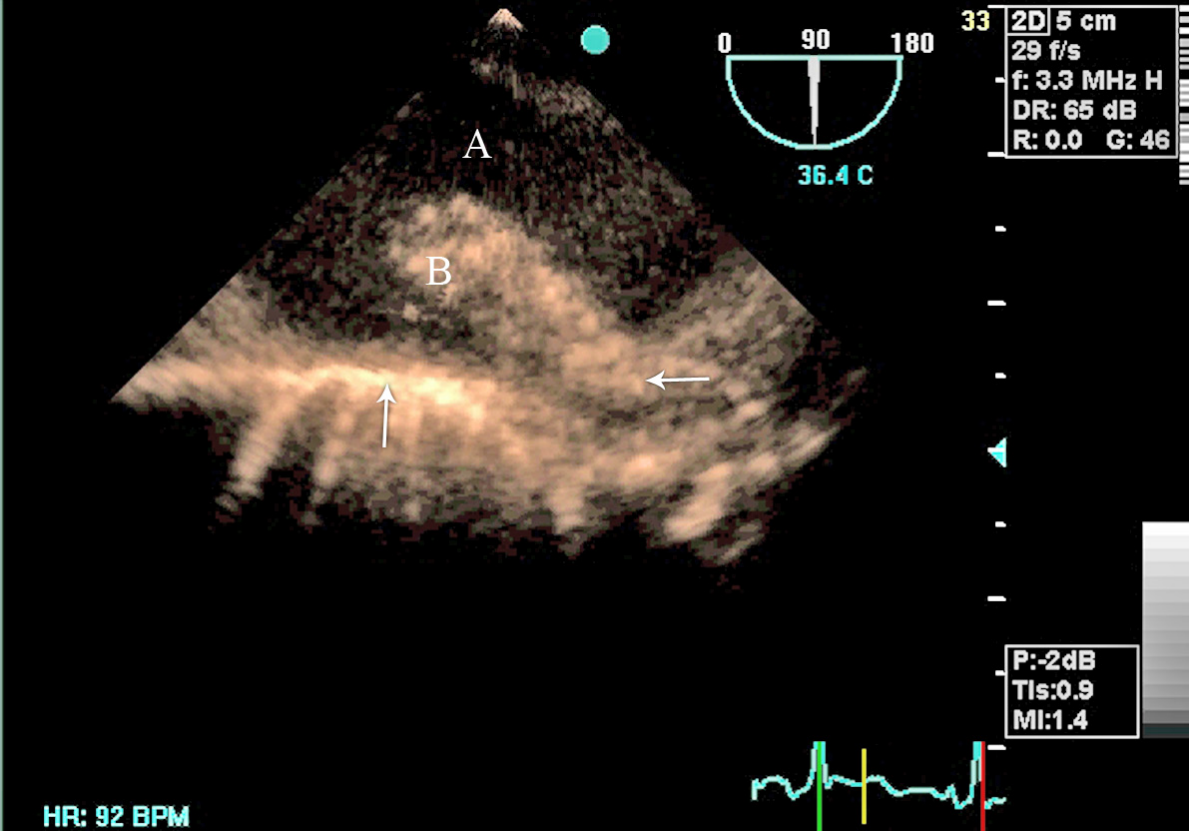
Transesophageal echocardiography (TEE) reveals a large mobile mass in the aortic arch. TEE is obtained at 90° angle and demonstrates the aorta and the aortic mass in the longitudinal view. Letter A shows the internal lumen of the aorta. Letter B stands for the atherosclerotic plaque. Horizontal arrow shows the attachment site of the atherosclerotic plaque to the aorta. Vertical arrow shows the normal adjacent aortic wall.

The entity of the above-mentioned aortic mass was further elucidated via transesophageal echocardiography (TEE). TEE revealed normal left ventricular size and function and extensive atherosclerotic changes in the aortic arch with a large hypermobile mass (Figure 2 and Figure 3, Movie 2 and Movie 3). No dissection flap was observed in TEE.

**Figure 4:**
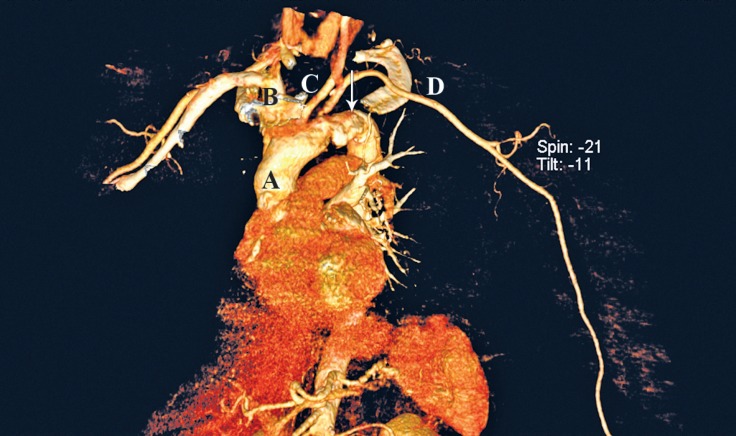
Computed tomography angiography of the aortic arch in the anteroposterior projection. In this 3D reconstruction of the major thoracic arteries, a large protruding mass is visible. Letters A, B, C, and D stand for the ascending aorta, innominate artery, left common carotid artery, and left subclavian artery, respectively. Vertical arrow shows a large atherosclerotic plaque in the aortic arch.

**Figure 5:**
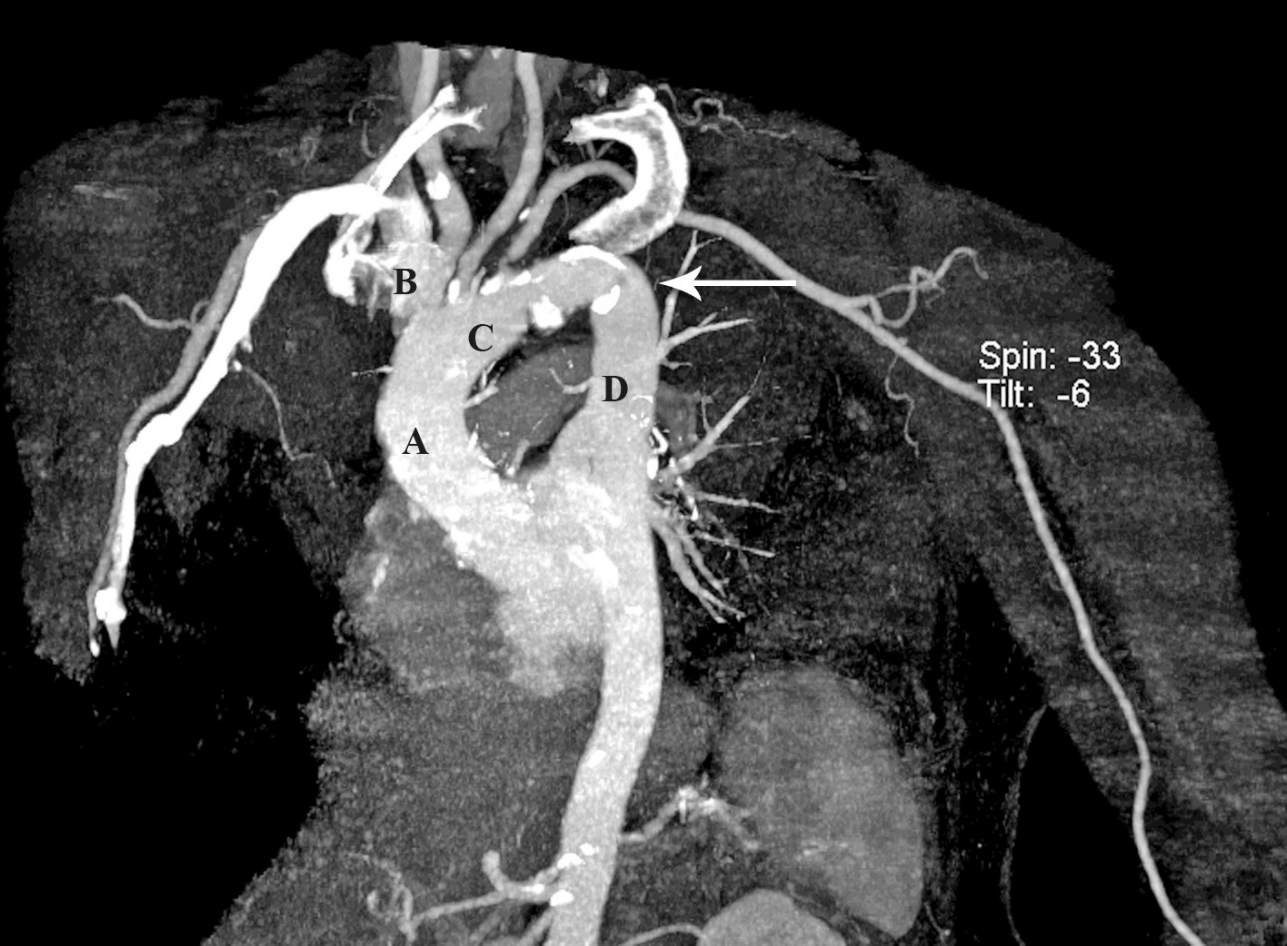
Computed tomography angiography of the aortic arch in the anteroposterior projection reveals calcified components of the aortic mass with density, similar to the adjacent bony structures. Letters A, B, C, and D stand for the ascending aorta, innominate artery, aortic arch, and descending thoracic aorta, respectively. Horizontal arrow shows calcified components of the aortic arch atherosclerotic plaque.

Computed tomography (CT) angiography of the entire aorta was requested. Dual-source multi-slice (2 × 128:256) CT angiography of the total aorta revealed a large floating mass (in favor of a thrombus) in the distal portion of the arch (Figure 4 and Figure 5). Additionally, there was a significant short segment left subclavian artery stenosis. Nonstenotic plaques were seen in the infrarenal aorta and common iliac arteries. The external iliac and common femoral arteries were calcified but without significant stenosis.

**Figure 6:**
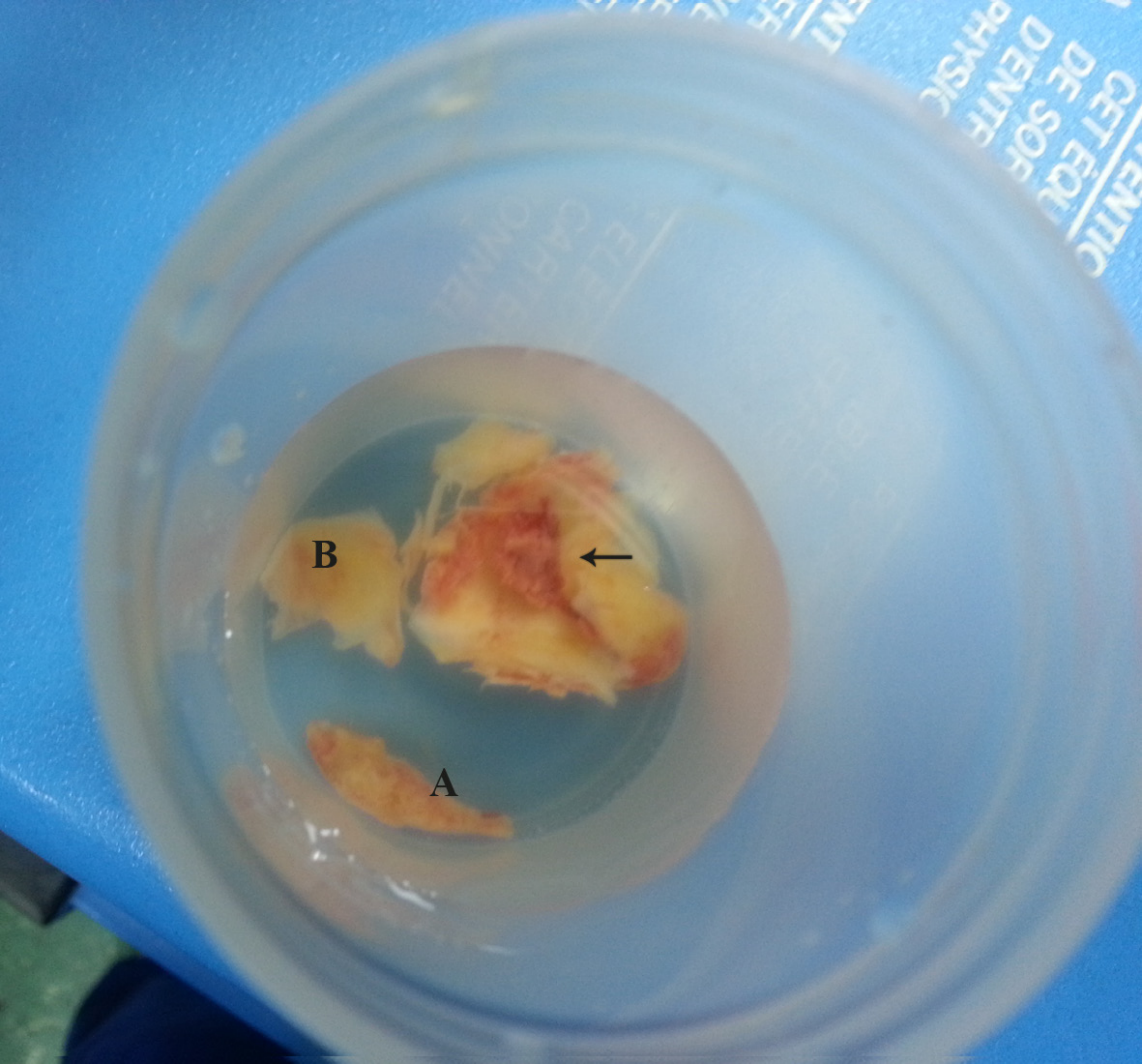
Atherosclerotic plaques resected during cardiac surgery. Letters A and B stand for atherosclerotic plaque particles after surgical resection. Horizontal arrow shows the large ulcerated atherosclerotic plaque.

The patient underwent coronary artery bypass grafting. The left internal mammary artery was not harvested due to the stenotic involvement of the left subclavian artery and saphenous vein grafts were anastomosed to the left anterior descending, obtuse marginal, and right coronary arteries. Intraoperative TEE confirmed a 15 × 7 mm mobile mass distal to the left subclavian artery. The aortic mass, which was apparently a large atherosclerotic plaque, was resected at the same session (Figure 6, Movie 4). The pathology report confirmed that the mass was a large atherosclerotic plaque. The patient recovered from the surgery without any adverse events and was discharged without any complications on the fifth postoperative day. During a 1-year follow-up after the procedure, no embolic events occurred.

## Discussion

Atheromatous disease of the aorta is a known indicator of vascular disease and is linked with ischemic stroke, peripheral embolization, and coronary events.

Atherosclerotic disease of the thoracic aorta is more prevalent in the elderly and patients with clinical coronary artery disease. Clinical data indicate that plaques with thickness equal to or more than 4 mm have a high risk for embolization. Plaques with a thrombus or a mobile component are usually referred to as complex atheromas.^6^

Patients with protruding aortic arch atheromas are at high risk for intraoperative stroke, significant and multiple morbidity, prolonged hospital stay, and death resulting from cardiac surgery. Aortic arch endarterectomy is strongly associated with intraoperative stroke.^7^

Sources of lower extremity and visceral arterial thromboembolism most commonly include the heart and proximal aneurysmal disease. Rarely, further workup of “cryptogenic” emboli will reveal a mural atheroma or thrombus of the descending thoracic aorta. Anticoagulation and open surgical thrombectomy have been the standard approach.^8^

Patients with mobile atherosclerotic aortic debris similar to our case are at the highest risk for catheter-related embolism during angiography. The strongest clinical predictors of atherosclerotic aortic embolism are advanced age and peripheral vascular disease. When atherosclerotic aortic debris is detected, particularly if the debris is mobile, substituting radial for femoral catheterization and avoiding the insertion of an intra-aortic balloon pump may lessen the risk of embolism.^9^

The gold standard for the diagnosis of atheromic plaques is CT angiography. However, in some patients, echocardiography from a suprasternal window can reliably visualize protruding aortic arch atheromas. Suprasternal window is a mandatory part of the classic TTE, but many operators omit this view due to patient overload and lack of time.

Atheromas are graded I to V. Grade I is minimal intimal thickening, grade II is extensive intimal thickening, grade III is sessile atheroma, grade IV is protruding atheroma, and grade V is mobile atheroma.^10^

Diffuse atherosclerotic disease of the aorta is becoming increasingly common as the population ages and the imaging modalities for its detection improve.

## Conclusion

Aortic atherosclerotic plaques are associated with high risk of embolic events, especially during catheter-based diagnostic modalities such as coronary angiography. Performing TTE before angiography with emphasis on the suprasternal window to detect aortic arch atheromas may alert the physician to perform the angiography more cautiously. Small sessile plaques could be treated conservatively by modifying the risk factors or the medication. Complicated plaques should be addressed more aggressively. In our case report, the plaque was mobile and ulcerated, which means that it was classified as a grade V lesion. Considering the concomitant coronary artery disease, excision of the plaque was reasonable during the coronary artery bypass graft procedure.

## Notes:

***This paper should be cited as: ***Aghajani H, Yazdani S, Ghafaripoor M, Shajari Z. Incidental Finding of a Large Mobile Aortic Arch Mass during Conventional Angiography. J Teh Univ Heart Ctr 2017;12(4):171-174.

## Notes:

***To watch the following videos, please refer to the relevant URLs:***

Movie 1.

http://jthc.tums.ac.ir/index.php/jthc/article/view/489/617

Movie 2.

http://jthc.tums.ac.ir/index.php/jthc/article/view/489/618

Movie 3.

http://jthc.tums.ac.ir/index.php/jthc/article/view/489/619

Movie 4.

http://jthc.tums.ac.ir/index.php/jthc/article/view/489/620
